# Informational Practices of Postacute Brain Injury Patients During Personal Recovery: Qualitative Study

**DOI:** 10.2196/15174

**Published:** 2019-11-12

**Authors:** Yamini Masterson, Erin Brady, Andrew Miller

**Affiliations:** 1 Department of Human-Centered Computing School of Informatics and Computing Indiana University-Purdue University Indianapolis Indianapolis, IN United States

**Keywords:** chronic illness, brain injury, disease management, mental health recovery, quality of life, data collection, personal health records, patient generated health data

## Abstract

**Background:**

The effects of brain injury, structural damage, or the physiological disruption of brain function last far beyond initial clinical treatment. Self-tracking and management technologies have the potential to help individuals experiencing brain injury in their personal recovery—helping them to function at their best despite ongoing symptoms of illness. However, current self-tracking technologies may be unsuited for measuring the interconnected, nonlinear ways in which brain injury manifests.

**Objective:**

This study aimed to investigate (1) the current informational practices and sensemaking processes used by postacute brain injury patients during personal recovery and (2) the potential role of quality-of-life instruments in improving patient awareness of brain injury recovery, advocacy, and involvement in care used outside the clinical context. Our objective was to explore the means of improving awareness through reflection that leads to compensatory strategies by anticipating or recognizing the occurrence of a problem caused by impairment.

**Methods:**

We conducted a qualitative study and used essentialist or realist thematic analysis to analyze the data collected through semistructured interviews and questionnaires, 2 weeks of structured data collection using brain injury–specific health-related quality of life instrument, quality of life after brain injury (QoLIBRI), and final interviews.

**Results:**

Informational practices of people with brain injury involve data collection, data synthesis, and obtaining and applying the insights to their lifestyles. Participants collected data through structured tools such as spreadsheets and wearable devices but switched to unstructured tools such as journals and blogs as changes in overall progress became more qualitative in nature. Although data collection helped participants summarize their progress better, the lack of conceptual understanding made it challenging to know what to monitor or communicate with clinicians. QoLIBRI served as an education tool in this scenario but was inadequate in facilitating reflection and sensemaking.

**Conclusions:**

Individuals with postacute brain injury found the lack of conceptual understanding of recovery and tools for making sense of their health data as major impediments for tracking and being aware of their personal recovery. There is an urgent need for a better framework for recovery and a process model for choosing patient-generated health data tools that focus on the holistic nature of recovery and improve the understanding of brain injury for all stakeholders involved throughout recovery.

## Introduction

### Background

Brain injury is a leading cause of death and disability around the world. In the United States alone, more than 3.5 million individuals sustain an acquired brain injury every year and an estimated 5.3 million Americans live with a related disability that causes lifelong challenges and reduced health-related quality of life (HRQoL) [1]. This is because of the complicated and longitudinal nature of recovery spanning months to several years affecting individuals through the interplay of physiological, psychological, social, and cultural factors [2-4]. That is, recovery extends far beyond clinical recovery [5], including *personal recovery*—the journey to a state where one is functioning at one’s best despite the ongoing symptoms of illness [6]. For example, though an individual does not exhibit routinely examined clinical symptoms such as unconsciousness or unresponsiveness [7], they might still experience brain fog limiting their ability to comprehend any form of communication or physical or cognitive fatigue severely limiting their level of work/leisure activity. The health care system currently focuses on clinical recovery and pays less attention to personal recovery, leading to reduced quality of life for individuals with brain injury.

### Factors Affecting Personal Recovery

Personal recovery depends on several factors such as the severity and nature of injury, premorbid health and lifestyle, demographics, social support, personality, and awareness [8-10]. Impaired awareness and lack of insight observed in individuals with brain injury is one of the major factors that leads to poor patient outcomes [11-13]. A lack of awareness causes neurological, cognitive, and personality/behavior limitations that are classified into [14] (1) intellectual, inability to understand that a function is impaired; (2) emergent, inability to recognize a problem when it is happening; and (3) anticipatory, inability to anticipate that a problem will occur as a result of a deficit.

The primary focus of rehabilitation for brain injury is to improve emergent and anticipatory awareness through *making sense of recovery,* leading to anticipatory and recognition compensation—implementing compensatory strategies by anticipating or recognizing the occurrence of a problem caused by impairment [14].

**Figure 1:**
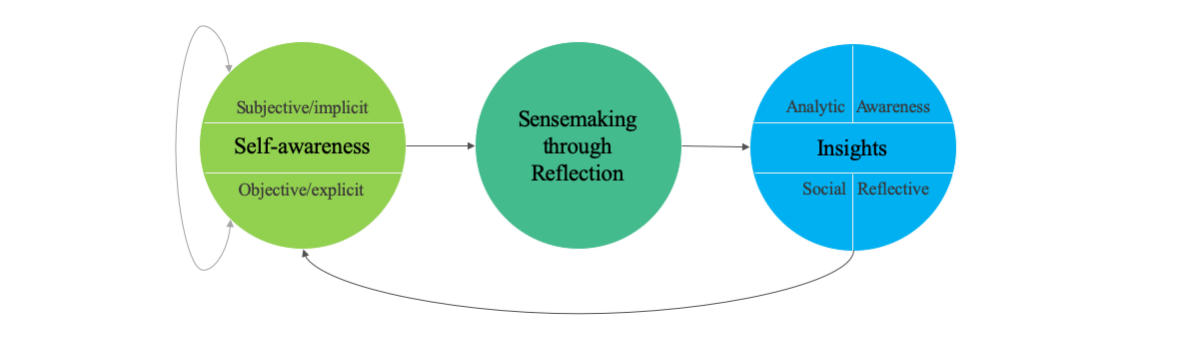
Process of sensemaking.

*Sensemaking* is “a process, prompted by violated expectations, that involves attending to and bracketing cues in the environment, creating intersubjective meaning through cycles of interpretation and action, and thereby enacting a more ordered environment from which further cues can be drawn” [15,16] (Figure 1). It is enabled by the 4 key dimensions of personal data collection—data, context, interaction, and insight [17,18]. *Reflection*, a method of sensemaking, involves awareness of discord between expectation and reality, leading to examining feelings and knowledge resulting in a new perspective [19]. One of the cognitive and affective skills required to engage in reflection is self-awareness (examination of how the situation and the individual affect each other) [19]. Of particular importance to brain injury recovery are the notions of subjective/implicit and objective/explicit awareness. Subjective self-awareness is an implicit process drawing from on abstract knowledge, whereas objective self-awareness is a conscious process involving synthesis of new and existing knowledge leading to reflection [20-22].

### Insights Through Sensemaking

An insight occurs intentionally or unintentionally through a process of cognition, understanding, and learning, followed by a change of views. Literature in personal informatics visualization lists 4 types of insights derived by individuals who collect and reflect on data about different aspects of their daily life [23]:

1. Analytic insights come from exploratory analysis and extrapolation and consist of the large or small eureka moments where a body of data comes into focus for a user.
2. Awareness insights come from maintaining awareness of a particular data stream that can give a sense of fluctuations in the data and its shifting patterns.
3. Social insight is the sense of understanding of a social group and one’s place in it.
4. Reflective insight is about oneself, the world, and one’s place in it.

### Tools for Measuring Personal Recovery

Personal recovery is measured by the extent of subjective well-being of a patient as overall well-being is the optimal goal of personal recovery [24]. The US Center for Disease Control and Prevention developed HRQoL for measuring well-being [25]. HRQoL provides valuable information on health status and treatment effects that could not be collected in any other way [26]. Moreover, self-rated health and higher self-awareness are long-term predictors of quality of life of brain injury patients [13,27].

Disease-specific scales have been developed to counter the methodological barriers to the adoption of HRQoL usage [28,29]. Truelle et al developed a traumatic brain injury–specific HRQoL—quality of life after brain injury (QoLIBRI)—with 37 items administered using six 5-point Likert scales: cognition, self, daily life and autonomy, social relationships, emotions and physical problems, and an overall scale [30]. These items help patients identify not only weaknesses but also their strengths and the impact on their own life situation.

### Assistive Technologies for Brain Injury

Early research for brain injury recovery focused on inpatient rehabilitation through game design and medium-technology tools for occupational therapy [31], physical therapy [32], and cognitive rehabilitation [33,34]. Self-monitoring and management have also been studied in an inpatient rehabilitation setting [35] and at home via aids for memory impairment using reminders [36], activity organizers [37], and social robots to help patients with loneliness [38]. Activity monitoring and pain assessment were used to improve awareness of self and activities that could lead to reinjury [39,40].

Many accessible technologies have been designed to address living with the individual symptoms of life with brain injury. A limited amount of this work has been conducted directly with individuals with brain injury, identifying strategies for dealing with things such as impaired memory formation [41] or difficulty with scheduling and planning [36,42]. A wider body of literature explores broader domains such as cognitive impairment, which impact people living with many different disabilities and their care providers and families [43,44]. However, there has been little research in the health informatics or human-computer interaction communities on the role of interactive tools to not only measure but also support personal recovery. The holistic understanding of personal recovery, which is more than the combination of individual symptoms, requires tools that take into account its interconnected, nonlinear, and iterative nature in facilitating sensemaking.

## Methods

To understand how individuals recovering from brain injury currently track and make sense of their personal recovery, we conducted a qualitative study to examine their sensemaking experience addressing 2 questions: (1) What are the current informational practices and sensemaking processes used by postacute brain injury patients during personal recovery? (2) How is QoLIBRI framework relevant to the process of personal recovery and what potential role can it play in patient context?

### Recruitment

We posted recruitment advertisements to online support groups for postacute brain injury survivors with a similar diagnosis and were contacted by 12 potential participants who were interested in the study. All members of the group underwent surgery for a growth in the same area of their brain.

Owing to the impacts of brain injury on cognitive functions, we conducted an initial screening phone call with each potential participant to explain the purpose of the research, answer any questions they had, and ensure their capacity to consent to the study. After explaining the study, we asked a series of questions to ensure that they understood the research procedure—for example, one of the questions asked: “What can you do if you decide after we start that you do not want to participate in the study?” An example of an acceptable answer would be “Tell you that I do not want to answer any more questions.” One participant was excluded as a result of not being able to answer the consent questions. In addition, 2 participants decided not to participate because of cognitive fatigue caused by taking the phone calls.

### Data Collection

**Figure 2:**
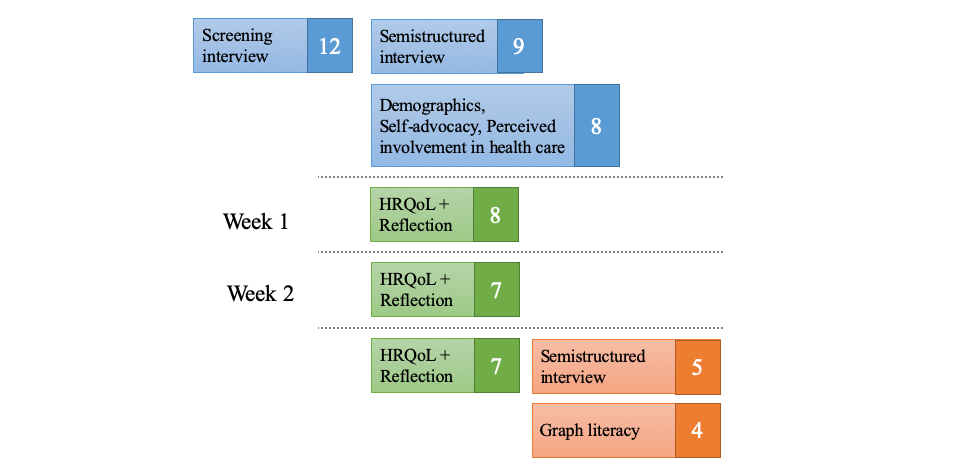
Study design and number of participants. HRQoL: health-related quality of life.

Data were collected through initial interviews and questionnaires, 2 weeks of structured HRQoL data collection, and final interviews and questionnaires. Figure 2 shows the timeline of data collection and number of participants in each activity.

All 9 qualified participants participated in an initial semistructured interview (protocol in Multimedia appendix 1) that lasted 60 to 75 min. This interview was based on recovery and rehabilitation measures and goals, tools for information tracking and synthesis, and clinical decision making [45].

Then, each participant answered questionnaires about (1) demographic information, (2) extent of self-advocacy, and (3) patient-perceived involvement in care (observing patient involvement) [46,47]. Participants were then asked to record HRQoL data reflecting on the previous week for 2 weeks, administered through Qualtrics. Furthermore, at the end of each week, the participants answered a questionnaire through which they reflected on the usefulness of the data they collected during the week. The tool was merely used as a data collection and participatory technology probe to study usefulness of the framework, and so, the participants could not observe trends with 3 weeks of data collection [48].

At the end of 2 weeks, they took a (1) final semistructured interview lasting 45 to 60 min (protocol in Multimedia appendix 2) and (2) a questionnaire for the researchers to gain an understanding of their graph literacy intended for a future study. The final interview discussed usefulness of QoLIBRI, reflection, and expectations from a data collection tool. Figure 2 shows the study design and number of participants who participated in each activity. Owing to scheduling and other health concerns, only 5 participants were able to take the final interview. We used a protocol that combined the initial and final interview for 1 participant because of availability. The details of activities done by each participant are listed in Table 1. The participants were paid US $20 each for interviews and US $20 for all the questionnaires. The Indiana University’s institutional review board approved this study.

**Table 1:** Data collected. All indicates that the participant completed the initial interview and questionnaires (I-1), 3 health-related quality-of-life questionnaires (HRQoL), a final interview (I-2), and a questionnaire on graph literacy (GL).

Participant ID	Data collected for study
P1	I-1^a^, 3 HRQoL^b^, GL^c^
P2	I-1, 3 HRQoL, GL
P3	All
P4	All
P5	I-1, 3 HRQoL, I-2^d^
P6	I-1, 1 HRQoL
P7	I-1, 3 HRQoL, I-2
P8	All
P9	I-1 and I-2 combined^e^

^a^I-1: initial interview and questionnaires.

^b^HRQoL: health-related quality-of-life questionnaires.

^c^GL: graph literacy.

^d^I-2: final interview.

^e^P9 did not complete the initial questionnaires and did the initial and final interview together.

### Analysis

Each interview was conducted over an audio call and was recorded. All the authors participated in a 6-phase essentialist/realist semantic thematic analysis to analyze the data from 14 interviews and 22 questionnaires [49]. We used both data-driven and theory-driven coding, identified themes using surface meanings of data, and used only participants’ experience and perspective for interpretation. To be faithful to the firsthand account of motivations and experience of individuals with brain injury, we present the significance of the patterns and their broader meanings and implications, mostly assuming a unidirectional relationship between meaning and language [50].

Data from participants P1 to P5 were collected between February and April 2017. We performed an initial analysis from May to July 2017, in which we learned that participants formulated their data collection and synthesis processes intuitively. To probe the intuitive nature of that process further, we revised the semistructured interview protocol and collected additional data from participants P6 to P9 from August to September 2017.

## Results

### Principal Findings

Informational practices of people with brain injury involve data collection; data synthesis; and obtaining, understanding, and applying insights to their treatment plans or lifestyles. Participants collected data through structured tools such as standard and customized spreadsheets, wearable devices, mobile apps, and calendar or daily log. As the frequency of symptoms decreased in postacute care, participants found the changes in overall progress more qualitative than quantitative in nature. So, they switched to unstructured tools such as journals, running notes on mobile devices, blogs, summaries from clinician appointments, patient portals, posts made on support groups, and inputs from caregivers.

Participants reported having insights about aspects of recovery such as symptoms; effect of medication, therapy, and lifestyle changes on symptoms; change in cognitive and physical abilities; changes in personality, identity, and social presence; and overall progress. They benefited from doing qualitative data collection alone; reflection through qualitative data collection improved self-awareness and thereby improved their understanding of their condition.

Although data collection helped participants summarize their progress better, they struggled with establishing a common language with health care providers. All participants highlighted a disparity in understanding of the impact of brain injury among different providers and the actual experience of a brain injury. Participants’ lack of a conceptual understanding made it challenging to know what to monitor in personal recovery and develop a template for collecting data. Owing to this, most of their challenges were centered around finding an effective tool, effort involved, and accuracy of data collection. In particular, we identified 2 key information needs for personal recovery from brain injury: education needs and awareness needs. On the basis of this, we proposed a potential design direction for personal recovery: structured qualitative data collection.

### Demographics

The participant pool consisted of 8 females and 1 male with a median age of 37.5 years. All our participants had more than high school education and identified as white.

- P1 and P3 had vocational/associate degrees; P2, P7, and P8 had bachelors’ degrees; P4, P5, and P6 had graduate degrees.
- P1 and P4 were employed for wages; P2 and P3 were students; P7, P8, and P9 were self-employed; P6 worked on freelance projects; and P5 was unable to work.

Although they underwent surgeries 1 to 4.5 years ago, symptoms presurgery lasted between 2 and 30 years because of reasons such as not being diagnosed, providers not being able to correlate symptoms with diagnosis, and not being able to find surgeons who were willing to perform the surgery. Participants reported high self-advocacy (education, mean=1.28; assertiveness, mean=2.21; and nonadherence when there is a disagreement, mean=1.9) and perceived involvement in care.

### Needs During Recovery

Our participants identified 2 major needs they experienced during their recovery process: *education* and *awareness* needs.

#### Education Needs

Participants expressed the need to be educated about the type of injury, risk factors, and prognosis associated with their brain injury. They found the lack of educational resources about the length of postacute care challenging to set expectations and plan rehabilitation. Often, they found it difficult to plan participation in instrumental activities of daily living (IADLs), social, and work activities without guidance. Negotiating accommodations at work and social activities also happen retroactively after experiencing difficulties. Yet, after the initial weeks of clinical recovery, identifying residual and new symptoms is a cognitive and logistical challenge. Lack of consistent follow-up care and rehabilitation programs added to the burden of self-advocacy, and participants reported having to *fight* to get appointments for addressing their postacute needs:

Because when I see [my doctor] for recovery, it’s only when I ask. It’s not like he has a program for me or anything.

P3, initial interview

The current sources of information described by our participants included (1) verbal information, paper, and online resources provided by health care providers; (2) online resources about health conditions; and (3) anecdotal information from health blogs and other brain injury survivors in support groups

To reduce overstimulation, exposure to infections, and hospital costs, all our participants underwent outpatient clinical recovery. During outpatient recovery, health care providers set expectations and gave instructions about the first few weeks of recovery but did not create awareness about the longitudinal nature of brain injury recovery. Participants understood, planned for, and worked on personal recovery through their own personal experiences and using information from anecdotal sources:

[My] neurosurgeon didn’t inform me of that. I find a lot of this through my own research as well as through my online support group and other people who have endured this. But honestly the answers don’t come from the doctors.

P4, initial interview

To compensate for this, participants reported using an online support group for setting expectations, crowdsourcing diagnoses by posting about their symptoms, and educating themselves about their new diagnoses. Similar to other online peer-to-peer communities for people with chronic illnesses [51], the collective knowledge of support groups helped set a roadmap, reminded them of symptoms to monitor for, and provided resources for seeking treatment:

You wind up going into a support group where you post the symptomology and people give you suggestions and lo and behold, it actually might hit. I had more success honestly with my support group in being directed and understanding certain things than I have with my doctors sometimes. I can’t emphasize that enough.

P4, initial interview

#### Awareness Needs

Participants’ awareness needs were twofold: (1) *personal* self-awareness to manage their condition and self-advocate and (2) *public* awareness to gain resources and services to support their recovery.

Lack of public awareness results in uncertainty of diagnosis and prognosis, lack of active follow-up by health care providers, lack of care coordination and planned care, and inadequate patient training to manage their illness. Without coordination between clinical care and rehabilitation providers, participants found it *dismissive* to have been discharged to outpatient recovery. They reported reduced quality of life as a result of recurring symptoms and impairment. Having to relearn some of their previous abilities to attain a new normalcy without guidance led to frustration, failure, and confusion in planning rehabilitation:

I did return to school in about 6 months after surgery...I couldn’t like remember what she would say and write it down before I forgot and also the overhead lights were really irritating to me which contributed as well to the migraine that I would get after class so I did drop out. I tried online and that didn’t work either because I am very sensitive to computer screens as well.

P2, initial interview

Apart from changing impairment, the burden of disease self-management reduced quality of life. Most participants reported cognitive impairment that changed based on physiological, psychological, and social factors. Having to change routines based on external and internal factors led to additional cognitive burden:

One day, I just wake up and I have head ache or pressure in my head and I can’t even do house work. Other day I wake up and I’m feeling good and I can do much more.

P3, initial interview

It was further complicated by secondary conditions and side effects of medication. Understanding the effect of these factors on their health and everyday life placed significant burdens on our participants as they were not trained for self-management of the disease. Thus, a major part of self-awareness is maintaining a biopsychosocial perspective of the disease:

...I remember when it was very difficult to think before surgery, I was very depressed. Hydrocephalus under control, and I can think clearly, then I can be optimistic.

P9, initial interview

To improve biopsychosocial awareness, participants used iterative self-experimentation. They experimented with various diets, sleep schedules, varying level of activity, etc, to gauge response. One participant introduced medication gradually to understand side effect and residual symptoms. They used clear hypotheses, schedules of intervention, and intuitive understanding of the effects. Thus, their self-awareness improved with multiple experiments and incorporating this understanding into planning everyday activities.

### Current Informational Practices

Informational practices of people with brain injury involve (1) data collection and (2) data synthesis, arriving at insights, and understanding insights through sensemaking.

#### Data Collection and Synthesis

Although they relied on imaging, physician evaluations, and diagnostic procedures such as blood tests and neuropsychological evaluations, participants also collected data that supplemented clinical indicators. We classified the data collection tools reported by the participants into the following:

- Structured: tools that specify data collected using a template or guidelines.
- Unstructured: tools that do not specify the type of variables or size of data.

Both the types of tools could be used to collect quantitative and qualitative data. For example, P8 reported collecting qualitative data using both structured and unstructured tools through a formalized process. She logged her symptoms every day and used a cognitive training app on her counselor’s suggestion. She then integrated these data into a weekly descriptive journal entry. In addition, she wrote a blog every month to update her caregiver group about overall progress.

Structured tools participants used included (1) standard and customized spreadsheets for tracking sleep patterns, medication, pain levels, symptoms, and triggers; (2) wearable devices for tracking sleep and heartrate; (3) mobile apps for tracking frequency, duration, and severity of symptoms; activity levels; and mapping factors that act as triggers to symptoms; (4) calendar or daily log for tracking frequency of symptoms; and (5) apps for cognitive training and tracking abilities (eg, Lumosity). The details of individual tools used by participants are listed in Table 2.

Participants reported that structured tools were most useful while in acute care and in the initial phases of recovery because of the higher volume, severity, and frequency of symptoms they experienced. Initial phases of recovery required being vigilant about the response to and side effects of medication. Structured data collection helped participants in noting patterns, identifying relationships between symptoms and triggers, and tracking adherence to prescribed medication and lifestyle changes:

I have a(n) iPhone app on my phone that I input what my pain level was for that day and I also chart anytime there was an increase in pain level and what I was doing prior to that what I think might have increased the pain level—weather forecast for the day stuff like that...It helps to kind of track the correlation between weather changes...It confirmed most of the triggers that we thought were triggers.

P2, initial interview

As the frequency of symptoms decreased in postacute care, participants found it unfruitful to collect structured data at the same frequency. Moreover, they found the changes in overall progress more qualitative than quantitative in nature. All participants reported switching to unstructured data collection tools and processes in postacute care. Owing to this, most participants reported extensive use of unstructured data for tracking personal recovery (P5, final interview: “But now, would be more like qualitative things like, how is my thinking, you know vision.”).

**Table 2:** Data collection tools used by participants.

Participant ID	Structured tools	Unstructured tools
P1	Fitbit for sleep and heart rate, Excel sheet for tracking sleep	None
P2	Mobile app for migraine triggers, medication journal, spreadsheet given by clinician to track pain level	None
P3	None	None
P4	None	Journal for tracking symptoms and medication
P5	Spreadsheet for symptoms and medication	Journal for symptom tracking, triggers, and medication, journal about overall progress
P6	Spreadsheet and calendar for medication, symptoms, and triggers, neuropsychological tests	Phone entries for symptoms and overall progress
P7	None	Notes by caregivers and patient for symptoms and medication, messages to health care provider through personal health records for overall progress
P8	Daily log of symptoms, Lumosity	Weekly log for symptoms—journal, monthly log for overall progress—blog
P9	None	None

Participants reported that making sense of unstructured data collection required more cognitive effort. Although the structured data collection process involved (1) collection of data by making note of stimuli and response and (2) synthesizing data using existing tools or mentally to obtain insights, the unstructured data collection process involved more steps, and each step required more effort. Participants reported (1) maintaining a constant awareness of progress, (2) preprocessing—synthesizing and reflecting, (3) collecting the data, and (4) synthesizing data mentally to obtain insights. As a result, participants reported that they did not have the capacity to collect unstructured data until later in the recovery process:

The reflection early on could cause some overstimulation and cause some of that frustration and anxiety in the early recovery stages.

P8, final interview

Participants used unstructured tools such as (1) journal entries for summarizing symptoms, triggers, medication, and overall progress; (2) running notes on mobile devices for changes in overall progress; (3) blogs for summarizing symptoms and overall progress; (4) summaries made from clinician appointments; (5) symptom communication with clinician through patient portals; (6) posts made on support groups about overall progress; (7) making mental note of unusual occurrences; and (8) inputs from caregivers.

For example, P8 reported integrating her symptoms from the previous week into a weekly descriptive journal entry. The other participants used unstructured data collection based on need. For instance, P6 used a running entry on her mobile device for noting anything unusual or changes in symptoms to report to her health care providers. Conversely, P7 integrated notes from clinician visits and messages sent on the patient portal reporting changes in symptoms to track her overall progress. Most participants reported using inputs from informal caregivers’ key to unstructured data collection and synthesis:

If I am near, someone like my husband would say your eye is swelling, you need to put ice on it and usually he is always spot on, my eye will swell prior to the onset of a migraine...

P2, initial interview

One participant used inputs from other patients on the support group to track progress and reported that sharing in these groups made it easier to observe trends in recovery:

...so I guess it’s another linear way of being able to track your progress and comparatively see where you might be. Again, you can’t compare apples to oranges because everybody is so different at recovery but at least you can get some sort of a baseline to see where you are.

P4, initial interview

All participants observed progress chronologically unless an event (travel, accident, new medication, or new diagnosis) required them to monitor the effect more closely. In such cases, participants switched to structured data collection while maintaining awareness of overall progress using unstructured data.

Few participants used formalized synthesis tools for unstructured data collection. They synthesized data mentally, gained an intuitive understanding of patterns, and compared trends chronologically to note improvements. Although many found this helpful, a few felt apprehensive about the subjective nature of this process:

My assumption of where I am and also having other people whoever is interacting with me the most within that timeframe, I ask their opinion and also, we compare it to where I was last month when we posted this...And so, it’s very subjective but it’s also worth saying here’s where I am now, and we can compare it to last month.

P8, initial interview

#### Insights Through Sensemaking

Participants described the process of having an insight as gaining an understanding of new information about postinjury self with respect to their environment that might lead to a change in their perspective of recovery:

To me, it doesn’t have to be very big...I have an understanding of something or an awareness of something that previously did not register with me which changed something about how I see something, so I have a greater insight.

P6, initial interview

Participants reported having insights about aspects of recovery such as (1) duration, frequency, severity, and triggers of symptoms; (2) effect of medication, therapy, and lifestyle changes on symptoms; (3) change in cognitive and physical abilities; (4) changes in personality, identity, and social presence; and (5) overall progress.

We identified some notable relationships between aspects of recovery and types of insights: (1) analytic and awareness insights are used to understand change in specific symptoms and effects of medication; (2) reflective insights correspond to changes in personality and identity; (3) social insights are useful in understanding how brain injury affected their social standing (P9, initial interview: “They forget about what you’ve been through, and they just think you’re useless.”) and where they stand in the process of recovery as compared with others (P1, initial interview: “I see a lot of people not being able to return to work for 6-12 months or not being able to return to work at all. I think I have been very lucky.”); and (4) social, reflective, and overall insights are usually specific to a context, whereas analytic and awareness insights are specific to data and interaction. On the whole, analytic and awareness insights emerged primarily from structured data, whereas unstructured data helped them gain all types of insights.

Having insights not only helped patients understand themselves better but also empathize with people with other conditions (P6, initial interview: “...I understand what other people are struggling with in their daily basis, issues from disability.”).

On the basis of the insights obtained, participants continually readjusted their concept of and expectations for recovery and learnt coping mechanisms:

Well the word “recovery” to me means that undetermined time period after having a major surgery in this case a brain surgery and you know it means resting and listening to your body...

P7, initial interview

I don’t have an end goal in mind. I learned that early on in my recovery.

P6, initial interview

Participants also made lifestyle changes so as to be flexible about their level of activity based on biopsychosocial factors:

For activity, on my calendar, I used to have, two or three things. If I’m going out anyway, I might as well do three things. Now, I learnt that I can’t do that. Let’s just do one thing and let’s go out five days a week or four days a week.

P3, final interview

This flexible approach enabled participants to seek accommodations at work and in social life. Having insights also empowered participants to self-advocate for required services and seek health care providers who are open to patient-centered care.

### Challenges

Although participants all engaged in tracking and reflection, they still faced significant challenges. Overall, 3 particular challenges emerged in analysis: (1) challenges in communicating their progress and setbacks with providers, (2) informational challenges deciding what to track and how to organize the data, and (3) emotional challenges.

#### Communication

Although data collection helped participants summarize their progress better, they struggled with establishing a common language with health care providers. All participants highlighted a disparity in understanding of the impact of brain injury among different providers and the actual experience of a brain injury. The participants, although not aware in the beginning, learnt to understand the circular causality of symptoms and view progress as a combination of quality of life and relief from symptoms. Health care providers, for the most part, have a biomedical perspective of recovery. Owing to this difference in perspectives, participants found it difficult to communicate with their providers:

When I logged my sleep patterns in excel for 6-7 months, my doctors refused to even look at it. It dissuaded me from carrying on.

P1, initial interview

Clinician-provided quality-of-life assessments, when available, were helpful in bridging this gap to a certain extent. However, the burden of translating patient-generated health data (PGHD) to clinician-provided instruments fell to patients, often resulting in duplicated effort:

So, every time I see my healthcare provider, they make me fill out their own surveys, so they are also tracking...I don’t see the results of that, but I’ll reference my information then as I’m filling out their survey.

P6, initial interview

Although participants highlighted the lack of health care provider mistrust in the usefulness of PGHD, these challenges also suggest inconsistencies in patient and provider objectives from and lack of provider awareness about the importance of quality-of-life data.

#### Informational

Participants’ lack of a conceptual understanding made it challenging to know what to monitor in personal recovery and develop a template for collecting data. Owing to this, most of their challenges were centered around finding an effective tool, effort involved, and accuracy of data collection. This may be partly because of the lack of tools that address personal recovery conceptually and customize for changing needs (P8, final interview: “I didn’t know that these were the areas, it would have been helpful to understand that all these areas would be major gaps in recovery.”).

Participants also faced challenges collecting data about symptoms and patterns because of the sporadic nature of many symptoms. Structured data collection is rigid and effortful in this respect. For instance, P2 found it challenging to note when the migraine started or what the triggers were if she woke up with a headache. Currently, structured tools do not allow mixed formats for either capturing subjective and objective components of an experience or visualizing integrated data.

As recovery progressed and participants collected more qualitative data, the frequency of insights decreased but the need for frequency of data synthesis increased. This is because of the usage of understanding of overall progress and day-to-day status in planning activities for the day or week. Maintaining biopsychosocial awareness and frequent synthesis is a major challenge of unstructured tools that none of the participants reported to have found a way around. Monitoring overall progress and specific symptoms and making frequent decisions based on this caused decision fatigue:

If I do (journaling) later in the day, brain fog ensues and then I just can’t think clearly and then I can’t really process the thought very accurately so that’s one of the challenges, fatigue, brain fog, clarity, and maybe knowing what the day brings, sometimes the day doesn’t bring much and in recovery some days are more active than others and sometimes the activity yields effect the next day pretty out of it, I have to kind of sit it out

P4, initial interview

Irrespective of the type of data collection and tools, brain injury patients deal with multiple sources and vast amounts of data. In spite of it being a continual process, the cognitive overhead of sensemaking cannot be underestimated (P6, initial interview: “I think my challenges are that, there is a lot of data to process, a lot of things are happening that I find hard to keep track of.”).

#### Emotional

Coupled with mental health issues, participants found coming to terms with their current level of progress and being reminded that recovery is still in progress emotionally challenging (P9, initial interview: “When I feel good, I don’t want to feel negative or go back on what’s emotionally challenging, my thoughts can be quite dark.”). Moreover, participants describe the process of recovery as a vocation rather than an activity because of the time it consumes:

I found my recovery was my actual job...So, it’s like all that was at least 50 hrs. a week—going to therapy and going endlessly to these appointments...I mean, I’m focused on my recovery and on what I can learn to help myself pretty single-mindedly.

P6, initial interview

### Relevance of Quality of Life After Brain Injury

#### Advantages

QoLIBRI provided participants with a conceptual framework for brain injury recovery. It served as an educational tool for understanding the areas that are affected by brain injury. Participants found all the sections of QoLIBRI relevant to areas of their recovery. Understanding recovery and using the instrument improved reflection and helped track progress in specific areas and monitor for changes in others:

...this assessment has really incorporated what my recovery is like. It expanded my knowledge of, it has targeted into areas that I walked through in my recovery...

P8, final interview

When participants were aware of change, data helped them with collecting evidence to validate it. Thus, QoLIBRI established a common language between the patient and health care provider, empowering the patients to communicate their insights and seek treatment (P3, final interview: “I think I’d probably see the doctor and explain that I’m not at where I’d like and see maybe if he gives some ideas to get further.”).

It helped with overcoming one of the challenges of qualitative data collection—having a structure for things to monitor (P8, final interview: “you can write things without saying something”). This awareness is useful in proactively monitoring for complications and being aware of changes before symptoms worsen. Participants reported reduced cognitive load from collecting data using QoLIBRI by externalizing information and not being preoccupied with monitoring and checking for changes throughout the day. Though it did not reduce the emotional challenges of recovery, having a better understanding helped them with being aware of the services and providers that could aid their recovery:

...not really thinking so much about my brain health and where am I at you know brain injury wise, I think that would bring it back to the forefront having those questions.

P7, final interview

#### Challenges

QoLIBRI is a quantitative instrument that collects discrete data and restricts the type and format of data that patients can collect. Reviewing Likert scale data makes it difficult to tell a story and meaningfully engage with it. The level of detail for each section might vary for each patient and stage in recovery. QoLIBRI is not designed for this level of customization. Although participants reported that using QoLIBRI enabled reflection, it did not accommodate recording and processing insights. Even if it were to provide a way of recording insights, maintaining biopsychosocial awareness by synthesizing data and insights remains a challenge.

#### Potential Role of Quality of Life After Brain Injury and Implications for Design

In its current state as a static instrument, QoLIBRI was most useful to participants as an educational resource and the framework as a reference for collecting data.

Although QoLIBRI is a starting place, there is an urgent need for a framework for recovery that overviews how symptoms change to guide both patients and health care providers more effectively through the process. Patients might have to customize the level of detail for each section or focus on certain parts of the tool for specific periods of time. A tool based on this framework would be more useful if it allowed capturing both qualitative and quantitative data. Although the template is disease specific, standardizing the language for all the health care providers involved in caring for a brain injury patient could improve patient participation in health care decision making.

This could then be useful for creating a process model for patients to choose appropriate tools throughout recovery. Using the model might be especially useful for testing hypotheses or changing the frequency of data collection. After participants switched to qualitative data collection, they did it at a lower frequency or in short bursts when symptoms changed. An important aspect for practice would be to integrate both of these into patient/caregiver education and health care provider training.

## Discussion

As our participants described to us repeatedly, recovery from brain injury extends far from the current biomedical understanding. Although prior studies demonstrated that incorporating quality-of-life measures in clinical decision making improved patient outcomes and well-being [52], our study shows that the current quantitative assessments for measuring quality of life are inadequate in understanding personal recovery. Our analysis highlighted the need for exploring personal health informatics beyond tools for collecting discrete measures. On the basis of our findings, we see opportunities for the areas of health care processes and personal health informatics to improve patient engagement in clinical decision making.

### Improve Conceptual Understanding of Recovery

The lack of conceptual understanding of recovery is a major barrier to reliable data collection and effective communication with health care providers. Research in chronic disease management shows that knowledge about disease prognosis is integral to disease management and greatly impacts patient outcomes [53,54]. Though knowledge exchange through peer support serves as an important component of patient education [51,55,56], formally addressing this begins with prioritizing health care processes and improving resources for patient education.

Our participants also highlighted the need for establishing a common understanding of and language for recovery. This could lead to similar patient and provider objectives from quality-of-life data, which is still a major barrier to integrating PGHD into clinical decision making [57,58]. We believe that this cannot be achieved without training health care providers to interpret PGHD and incorporate that into clinical processes and providing patients with tools that are intuitive and effective in their context [59]. Educating health care providers about the type of data and methods patients use for self-management might shift the focus from disease to illness, thereby providing a biopsychosocial perspective for clinical context and establishing a common language for health care providers and patients to communicate [60,61].

Participants in our study benefited from qualitative data collection as it implicitly invoked reflection, which in turn improved self-awareness and understanding of their condition:

So, it is helpful in being able to see, although it is glacial, some of the areas improved. When you write it down, to an extent, it is cathartic, you are releasing some of the stuff that has been going on.

P4, final interview

However, this was effective and useful when coupled with a conceptual understanding of brain injury recovery. To address this, we suggest using qualitative data collection in combination with the quality-of-life framework to enable reflection about personal recovery. This provides a template to follow progress while being flexible to customize for changing needs through recovery (eg, additional tracking for quality of sleep). Developing tools that allow *structured qualitative data collection* could be useful not just for brain injury but for any illness that requires self-management and lifestyle changes based on inferences from sensemaking [21,62,63]. Our current and future work involves studying how to design technology-agnostic structured qualitative data collection tools:

...what kind of format to put it in, especially subjective to where you are in the recovery process, you might not even be able to process well enough if you are writing a paragraph and then again, not really, without direct questions, hard to measure.

P8, final interview

### Improve Biopsychosocial Awareness Through Making Sense of Personal Recovery

Participants managed their illness and made decisions about activities of daily living and IADLs based on their biopsychosocial awareness [61]. In spite of it being challenging, they reported sensemaking through reflection as a continual, iterative, abstract, and subconscious process in illness management. In agreement with the literature, they engaged in a nonlinear process consisting of implicit self-awareness, description (recognition, recollection, and providing a comprehensive account of the event), critical analysis (“examining the components of a situation, identifying existing knowledge, challenging assumptions, and exploring alternatives”), synthesis (integration of new and existing knowledge to solve problems and predict likely consequences of actions), and evaluation (making judgements about the value of something) [19]. When used intuitively and iteratively, participants did not find the need to engage in every phase and could make a mental shift to analysis and synthesis. Owing to it becoming a second nature, they did not perceive insights gained through such a process as *aha* moments.

Conversely, they reported cognitive load from engaging in explicit self-awareness because of the need to consume external and detailed information. Moreover, similar to individuals who self-manage other chronic illnesses [64,65], our participants agreed that a lack of tools that enable sensemaking of multiple types of data increased the burden of having to consolidate and interpret data from different sources. Yet, the boundaries between implicit and explicit self-awareness were blurred (P3, final interview: “Something I’ve always known”). This could be because of 3 reasons:

1. The qualitative nature of data that participants synthesize mentally in spite of using a tool.
2. Improved subjective self-awareness with the need for self-advocacy.
3. Unavailability of tools for integrating and synthesizing different sources of data.

Participants’ insights were complicated and could be characterized as more than one type. In a few instances when the insight was too messy and abstract, the current framework was inadequate to classify it. Moreover, insights also changed based on the frame of reference. For instance, 1 participant changed her frame from reflective to social to gain a different insight:

you might think it as a detriment that now you have to rely on other people. But if you think about it in another way, well how you are being more social, and you may be developed that skill a little bit and may be that helps other people as well...

P5, final interview

This shows how the biological, psychological, and social aspects of an individual’s life are interconnected and interleaved with the overall wellness and so designing tools for sensemaking with this perspective would improve usability and integration into patient context [60,66].

### Involve Networks of Illness Self-Management

Communicating current levels of progress with stakeholders such as informal caregivers, family and friends, workplace, and health care providers is an integral component of illness self-management. Although informal caregivers are responsible for communication during clinical recovery, brain injury patients handle and make sense of data in postacute recovery that usually involves transition to self-management. This requires the patients to articulate their interpretation based on the stakeholder they are communicating with [60].

Informal caregivers, family, and friends need to be informed about current challenges, assistance required, and accommodations they need to make to ease rehabilitation into personal life. Patients need to evaluate their abilities and impairment and communicate this with the workplace for accommodations. Conversely, health care providers need to be informed about the current symptoms and services required by the patient to attain the desired quality of life. Thus, the same interpretation needs to be articulated as different constructs based on the scenario.

Designing tools for networks of illness self-management is essential to the integration of such tools into patient’s lives [67]. Leveraging constructs such as quality of life is helpful for (1) translation of PGHD into clinical variables, (2) providing a common language between patients and providers, and (3) shared decision making [60,68]. Improving communication could also reduce the general mistrust in the validity of PGHD and encourage incorporating it into clinical processes. Subjective measures shed light on the biopsychosocial nature of diseases and enable health care providers to empathize and provide patient-centered care.

### Limitations

In this paper, we presented the perspective of postacute brain injury patients recruited from an online support group for a specific type of brain injury.

Although the homogeneity of the condition presented common challenges, homogeneity of demographics such as socioeconomic status, race, and gender is a limitation of the study. As our participants had a higher education and socioeconomic status than the national average, they might experience lower resource scarcity and higher environmental stability, and thus have more opportunity for focusing on future needs, critical thinking, and self-reflection [69]. In addition, as participants were recruited from a text-based online support group, they may have fewer symptoms relating to communicative or social ability than other people with brain injury. Therefore, this might not be the most representative sample of individuals with brain injury. Yet, their challenges such as gaining biopsychosocial perspective, emotional burden, and lack of knowledge about disease are very similar to individuals with chronic conditions and multiple comorbidities [70,71], so we believe the findings of this study might be applicable to self-management of chronic illnesses in general.

Our participants were recruited remotely to ensure flexibility in participation. In spite of that, we still witnessed a high participant dropout rate stemming from health challenges and other complications of life after brain injury. General disbelief in researchers and fear of changing social dynamics in the group also creates a reluctance to participate in health research. The perspective of health care providers is out of the scope of this study.

### Comparison With Prior Work

Self-tracking and management technologies have potential to help individuals experiencing personal recovery to function at their best despite ongoing symptoms of illness. However, current self-tracking technologies designed for a general audience may be unsuited for measuring the interconnected, nonlinear nature of personal recovery from brain injury. Moreover, little is known about the potential opportunities and barriers for information systems to support personal recovery from brain injury. Furthermore, an understanding of this also has implications for the study of self-management of other chronic illnesses.

### Conclusions

We conducted a qualitative study for understanding the current informational practices and sensemaking processes used by postacute brain injury patients during personal recovery and the relevance of the QoLIBRI framework in patient context. Our participants highlighted the lack of conceptual understanding of recovery and lack of tools for making sense of their health data. On the basis of this, we discussed improving the validity of PGHD. Our research has implications for policy, process, and technology design involving all the stakeholders of health care to democratize clinical decision making.

## Appendix

Multimedia Appendix 1 Initial interview - semistructured protocol guide.

Multimedia Appendix 2 Final interview - semi structured protocol guide.

## Abbreviations

HRQoL: health-related quality of life
IADLs: instrumental activities of daily living
PGHD: patient-generated health data
QoLIBRI: quality of life after brain injury
