# Supplementary material for: Informational Practices of Postacute Brain Injury Patients During Personal Recovery: Qualitative Study
Source: J Particip Med. 2019 Nov 12;11(4):e15174. doi: 10.2196/15174 (PMC7434071; doi:10.2196/15174)
Supplement: Multimedia Appendix 1 [file jopm_v11i4e15174_app1.pdf]

Initial Interview

General

1. Please explain your condition briefly.
2. How long did you have symptoms related to this condition?
3. What kind of symptoms do you still have?
4. Which of these symptoms were not expected by you or your health care provider?
5. Are you currently being treated for these symptoms?
6. Do you have any symptoms that you are not being treated for?
7. At this point, are there any symptoms that you think might not be resolved?

Recovery

8. What does recovery mean to you?
9. How would you describe your recovery process so far?
10. What did your doctors tell you about your expected course of recovery? Did any of them explain the expected course of recovery?
11. How is your recovery as compared to the expected course?
12. What is the clinical assessment of your condition?
13. How is your subjective well-being as compared to the clinical assessment of your condition?
14. Why is this different? How do you know this is different?
15. Did you express this to your doctors? What was her response/suggestion?
16. How are your doctors helping you with this?
17. What else do you think your doctors could do to help with your recovery?

Rehabilitation

18. Did you have to make any changes to your life as a result?
19. What do you see as your problems resulting from your injury?
    i. Physical abilities (e.g. movement of arms and legs, balance, vision, endurance)?
    ii. Memory/confusion?
    iii. Concentration?
    iv. Problem-solving, decision-making, organizing and planning things?
    v. Controlling behavior?
    vi. Communication?
    vii. Getting along with other people?
    viii. Has your personality changed?
20. What is the main thing you need to work on/would like to get better?
21. To what extent do you think you rehabilitated into your life?
22. What measures did you take to enable recovery and rehabilitation?

Tracking

23. How do you manage your symptoms?
24. How do you keep track of your symptoms and recovery? (pre to post-op, how did symptoms change qualitatively and quantitatively – how do you know?)
25. What tools do you use to track your recovery – personal health records, personal memoirs, blogs, appointments with psychologists etc.? How do they help?
26. How do you understand the information from these tools holistically?

27. How is this information useful?
    i. For your understanding
    ii. For communication with healthcare providers
    iii. For communication with family/friends/caregiver group
    iv. For communication at workplace
    v. For communicating needs at a public place
    vi. Is there anybody else you share this with?
28. What kind of insights did you gain so far?
29. How did the insights help?
30. What do you understand about the status of your recovery from this information?
31. What are your current challenges with these tools?
32. What else do you need to help you track your recovery?
33. If you could come up with a tool that would help you in this process, what would it look like?

Participation in clinical decision making

34. How do you communicate these insights to your healthcare provider?
35. Would you show this data to your healthcare provider? (Why not?)
36. What was his/her response?

Recovery goals

37. What do you hope to achieve in the next 6 months?
38. What are your recovery goals for the next 6 months?
39. Do you think your brain injury will still affect your life in 6 months' time? How?
40. Are you satisfied with your current level of recovery?
41. Do you feel productive in your life and hopeful about your future?
