# Supplementary material for: Informational Practices of Postacute Brain Injury Patients During Personal Recovery: Qualitative Study
Source: J Particip Med. 2019 Nov 12;11(4):e15174. doi: 10.2196/15174 (PMC7434071; doi:10.2196/15174)
Supplement: Multimedia Appendix 2 [file jopm_v11i4e15174_app2.pdf]

Final Interview

Events

1. How has your health been in the past month?
2. Did anything out of the ordinary happen?

Symptoms

3. What symptoms did you experience in the past couple of weeks?
4. Do you know what triggered or affected the symptoms?

Sections of QOLIBRI (We look at their data and talk specifics)

5. How is HRQoL different from the other tracking you do?
6. How was each section of the questionnaire helpful – thinking abilities, emotions, independence, and physical problems? What kind of data did each of these sections help you capture?
7. What did you understand from capturing this data?
8. (Referring back to the symptoms) Did logging this information help you reflect on what affected your symptoms?
9. If not, what do you think would help? What other data would you like to collect?
10. If yes, what actions did you take because of the reflection you obtained from recording your health data?

Overall

11. How would you describe your experience of taking quality of life assessments?
12. Could you comment about the frequency of the assessments? Would it help if the assessments were more or less frequent?
13. Could you comment on your experience of data collection?
14. Is there any other way of data collection that would be easier?
15. What are the advantages to logging the quality of your health?
16. What are the disadvantages to logging the quality of your health?
17. What are the challenges of logging the quality of your health?
18. Is there anything else about the assessments you would like to share with us?

Reflection

19. (Referring to the initial interview) How does HRQoL match your perspective of recovery?
20. How does HRQoL data match your current stage of recovery?
21. How was reflecting on the assessment helpful to understanding your recovery?
22. What are the advantages to reflecting on the assessment?
23. What are the disadvantages to reflecting on the assessment?
24. What did you learn from reflecting on the assessment that you didn't from taking the assessment?
25. How would this help other patients with brain injury?
26. Is there anything else you would like to share with us?

Expectations

27. What did you expect to understand from participating in the study and collecting this information?
28. Was the questionnaire able to give you those insights? How/why not?
29. If you could personalize the assessment, what information would you collect?
30. If you could think of a tool that could help you understand your recovery and communicate it with your health care provider, what would it look like?

Study

31. How did you feel about participating in this study?
32. How did participating in this study help you?
33. What was the most difficult part about participating in this study?
34. Is there anything else you’d like to tell us about this research/ design?
